# Supplementary material for: Impedimetric and Plasmonic Sensing of Collagen I Using a Half-Antibody-Supported, Au-Modified, Self-Assembled Monolayer System
Source: Biosensors (Basel). 2021 Jul 8;11(7):227. doi: 10.3390/bios11070227 (PMC8301786; doi:10.3390/bios11070227)
Supplement: Supplementary file 1 [file biosensors-11-00227-s001.zip › biosensors-1245836-supplementary.pdf]

biosensors

MDPI

Article

Impedimetric and Plasmonic Sensing of Collagen I Using a Half-Antibody-Supported, Au-Modified, Self-Assembled Monolayer System

Abstract: This research presents an electrochemical immunosensor for collagen I detection using a self-assembled monolayer (SAM) of gold nanoparticles (AuNPs) and covalently immobilized half-reduced monoclonal antibody as a receptor; this allowed for the validation of the collagen I concentration through two different independent methods: electrochemically by Electrochemical Impedance Spectroscopy (EIS), and optically by Surface Plasmon Resonance (SPR). The high unique advantage of the proposed sensor is based on the performance of the stable covalent immobilization of the AuNPs and enzymatically reduced half-IgG collagen I antibodies, which ensured their appropriate orientation onto the sensor's surface, good stability, and sensitivity properties. The detection of collagen type I was performed in a concentration range from 1 to 5 pg/mL. Moreover, SPR was utilized to confirm the immobilization of the monoclonal half-antibodies and sensing of collagen I versus time. Furthermore, EIS experiments revealed a limit of detection (LOD) of 0.38 pg/mL. The selectivity of the performed immunosensor was confirmed by negligible responses for BSA. The performed approach of the immunosensor is a novel, innovative attempt that enables the detection of collagen I with very high sensitivity in the range of pg/mL, which is significantly lower than the commonly used enzyme-linked immunosorbent assay (ELISA).

Keywords: collagen type I; 4,4′-thiobisbenzenethiol; nanogold; electrochemical impedance spectroscopy; surface plasmon resonance; half antibody; medical diagnostic devices

3. Results

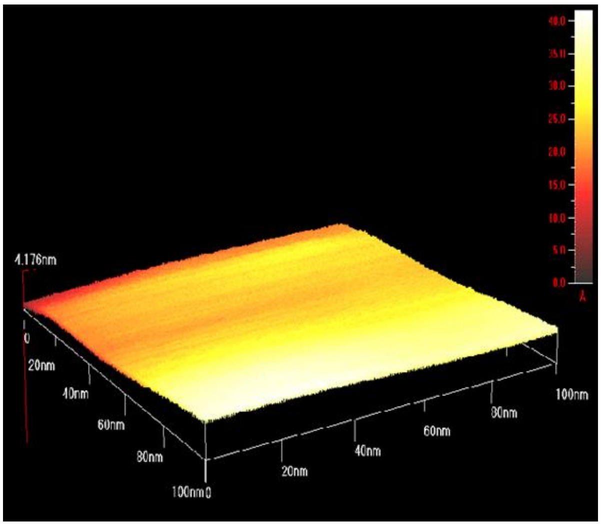

Figure S1. AFM 3D image (0.1 by 0.1 μm) of mica substrate coated with thin film of Au (111).

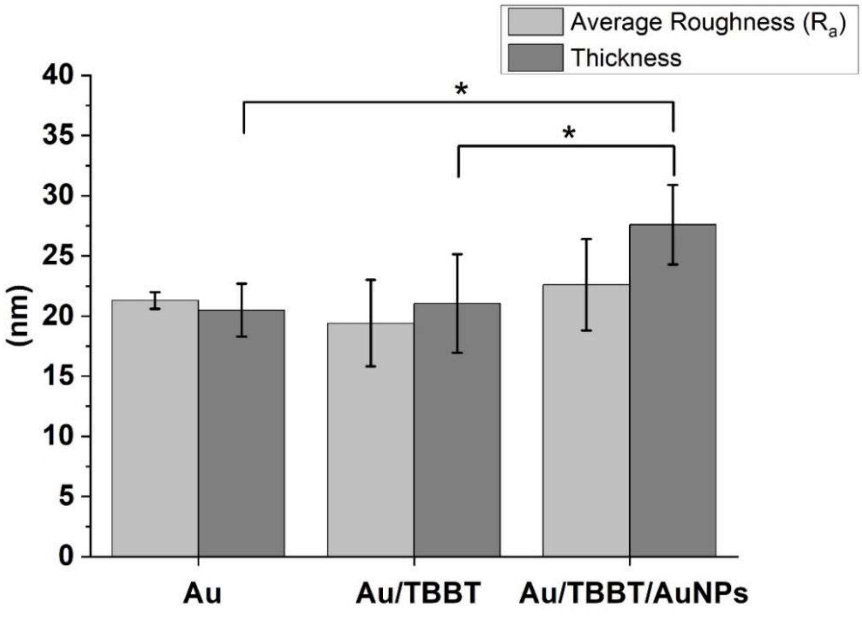

Figure S2. Results of the average roughness (Ra) and thickness for bare gold electrode surface (Au), after modification of TBBT (Au/TBBT) and after immobilization of the gold nanoparticles (Au/TBBT/AuNPs)**.** The calculation of the significant differences ($p<0.05$) was performed by one way ANOVA post-hoc Tukey Test and it was marked by the bars with star (*). .

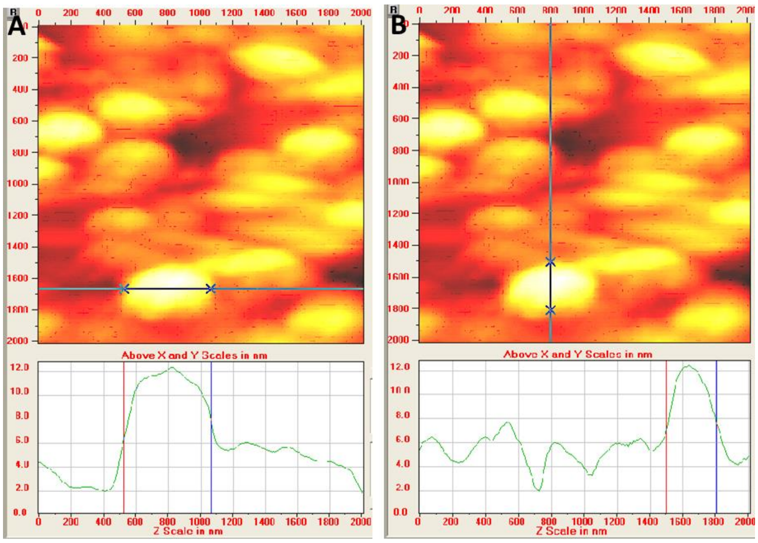

Figure S3. AFM 2D image of AuNPs modified gold surface (mica substrate coated with thin film of Au (111)). Representative images of A - length and B - breadth measurements of one particle.

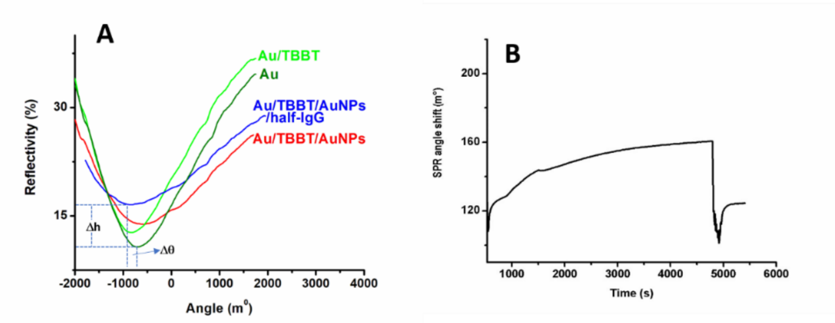

Figure S4. (A) Plot of reflectivity vs angle shift of surfaces with the change of reflectivity percentage for (i) Au, (ii) Au/TBBT (iii) Au/TBBT/AuNPs and (iv) Au/TBBT/AuNPs/half-IgG surface in suffer **(B)** SPR confirmation of half-IgG immobilization on the surface of Au/TBBT/AuNPs in PBS buffer at pH 7.

The Figure 4SA represents the change in the reflectivity angle with the each step of electrode fabrication. The study on plasmon dip variation in the process of fabrication of LIP/GO/4-ATP/Au SPR surface reveals useful information on the plasmonic behavior of the SPR surface and the depth of the surface layer. It demonstrates the shift of SPR angle with the change in refractivity percentage for (i) Au, (ii) Au/TBBT (iii) Au/TBBT/AuNPs and (iv) Au/TBBT/AuNPs/half-IgG surfaces. Self-assembly of insulating TBBT on Au SPR surface is confirmed by an angle shift and reduction of plasmon dip of the peak. Thus fabricated insulating TBBT change the refractive index of the Au/TBBT SPR surface. After the attachment of AuNPs broadening of the peak and reduction of plasmon dip is noticed. Further, a shift of response angle, broading of the peak and decrease in plasmon dip indicates immobilization of insulating half-IgG onto the Au/TBBT/AuNPs surface.
